# Supplementary material for: Effect of Site and Phenological Status on the Potato Bacterial Rhizomicrobiota
Source: Microorganisms. 2022 Aug 29;10(9):1743. doi: 10.3390/microorganisms10091743 (PMC9501399; doi:10.3390/microorganisms10091743)

Effect of site and phenological status on the potato bacterial rhizomicrobiota

Lisa Cangioli[1], Ada Daniela Baldi[2,*], Camilla Fagorzi[1], Marco Mancini[2], Simone Orlandini[2], Francesca Vaccaro[1], Alessio Mengoni[1,*]

[1] Department of Biology, University of Florence, via Madonna del Piano 6, 50019 Sesto Fiorentino, Italy
[2] Department of Agriculture, Food, Environment and Forestry (DAGRI), University of Florence, Piazzale delle Cascine 18, 50144 Firenze, Italy
* correspondence: A.D.B., ada.baldi@unifi.it); A.M., alessio.mengoni@unifi.it

Supplementary information file

Dataset S1: Table reporting the amplicon sequence variants (ASVs) detected, their taxonomic assignment and occurrence; .csv file.
Table S1: Table containing, sample codes, locality, phenological stage, fast1 file name, the number of total input reads from fastq files and the number of reads that passed the filtering parameters; .xlsx file.
Table S2: Table containing the physicochemical features of soil samples; .xlsx file.
Table S3: Table of Good's coverage values. Good's coverage was estimated on the amplicon sequence variant (ASV) table for each sample after removing chimeras; .csv file.
Table S4: List of richness, diversity and evenness indices; .csv file.
Table S5. Results of Permutational Multivariate Analysis of Variance (PERMANOVA) test performed on alpha diversity indicates; .csv file.
Table S6: List of p values calculated through a Permutational Multivariate Analysis of Variance (PERMANOVA) test performed on the phyloseq object after a centered log ratio (CLR) transformation; .csv file.
Table S7: Results of DeSeq2 on ASVs mostly differing among localities; .csv file.
Table S8: List of all identified metabolic pathways present in the taxa retrieved in the bacterial community; .xlsx file.

Figure S1: Climatic conditions. The cumulated growing degree days (GDD; °C) (top row) and the cumulated precipitation (mm) (bottom row) are reported. Results were calculated on a monthly basis (April-August) (left panel) and for the entire growing season (from sowing to maturity) (right panel).
Figure S2: Rarefaction curve of all soil samples. The x axis indicates the number of sequences found for each sample, while the y axis indicates the species richness.

Figure S1. Climatic conditions. The cumulated growing degree days (GDD; °C) (top row) and the cumulated precipitation (mm) (bottom row) are reported. Results were calculated on a monthly basis (April-August) (left panel) and for the entire growing season (from sowing to maturity) (right panel).

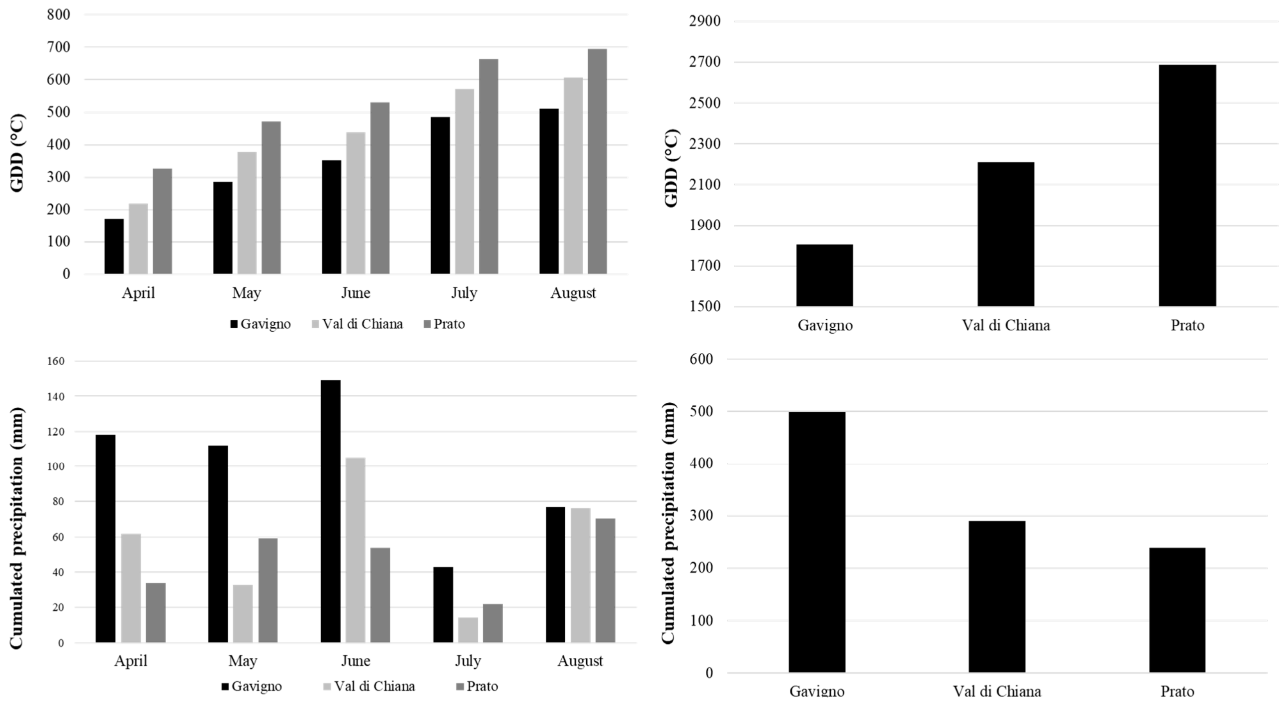

Figure S2: Rarefaction curve of all soil samples. The x axis indicates the number of sequences found for each sample, while the y axis indicates the species richness.

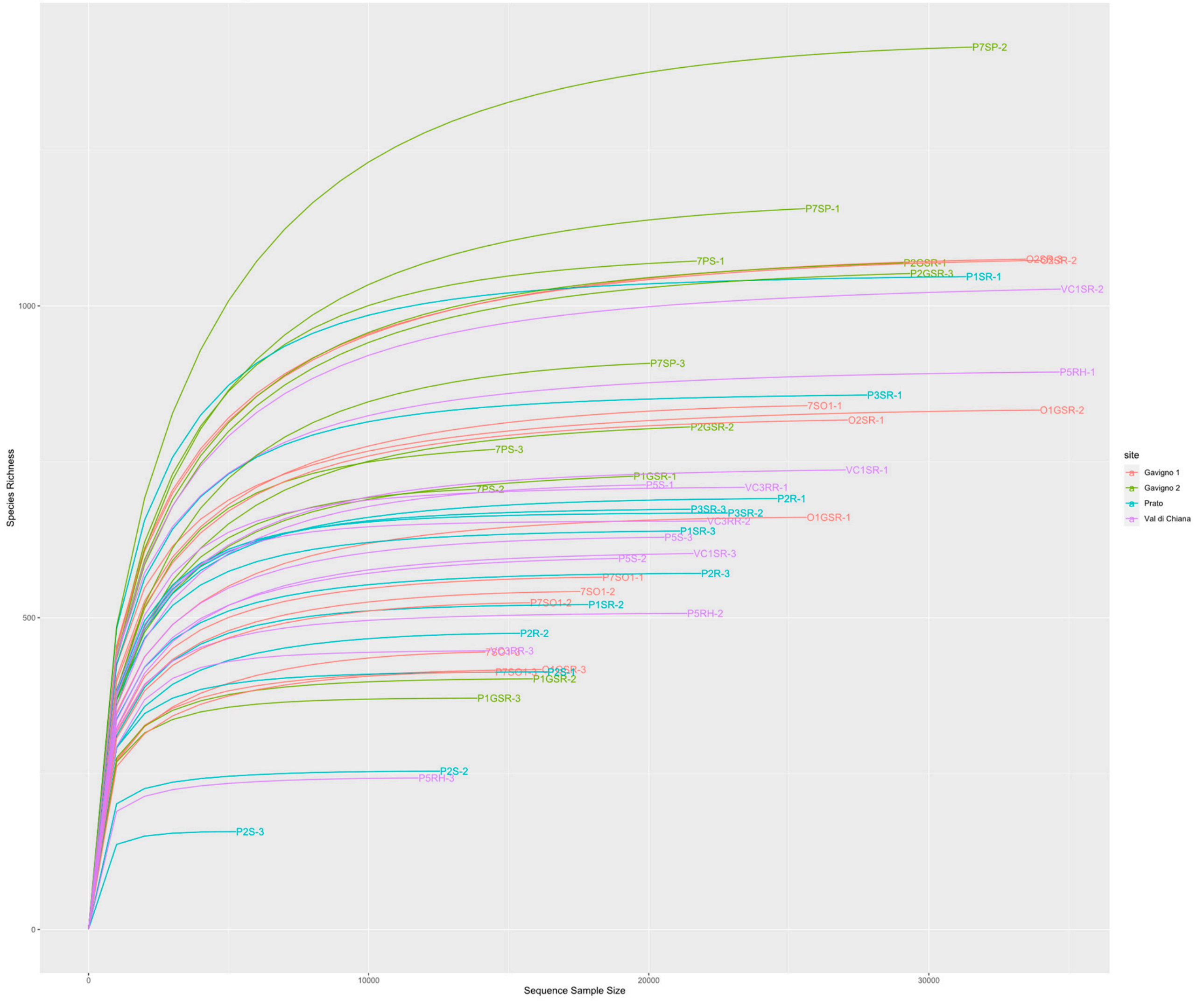

Supplement: Supplementary file 1 [file microorganisms-10-01743-s001.zip › Cangioli_patata_Suppl_Info_submitted.pdf]
